# Antioxidant Potential of *Schistocerca gregaria* (Orthoptera: Acrididae) Extract: HPLC Analysis, Molecular Docking, and Bioassays

**DOI:** 10.1002/cbdv.71685

**Published:** 2026-09-23

**Authors:** Magda M. Aboelmahasen, Reem M. Khorshed, Said M. Said, Shaymaa H. Mahmoud, Islam M. El‐Garawani, Heba M. R. Hathout

**Affiliations:** ^1^ Zoology Department Faculty of Science Menoufia University Shibin El Kom Egypt; ^2^ Zoology Department Faculty of Science University of Sadat City Sadat Egypt; ^3^ Locust and Grasshopper Department Plant Protection Research Institute Agricultural Research Center, Dokki Giza Egypt; ^4^ Natural Resources Department Faculty of African Postgraduate Studies Cairo University Giza Egypt

**Keywords:** antioxidant activity, biosafety, cytotoxicity, molecular docking, phenolic compounds, *Schistocerca gregaria*

## Abstract

Insects are emerging as a sustainable source of natural products in alternative and complementary medicine. This study evaluated the in vitro antioxidant activity and cytotoxic effects of methanolic extract of *Schistocerca gregaria*. Antioxidant activity was evaluated in the second, third, fourth, and fifth nymphal instars of *S. gregaria*. The results revealed that fifth nymphal instar exhibited markedly higher antioxidant capacity than the earlier instars with IC_50_ of 27.73, 74.71, and 34.32 µg/mL in the DPPH radical scavenging assay, FRAP and ABTS system, respectively for fifth nymphal instar of *S. gregaria*. Further characterization of the fifth nymphal instar methanolic extract was accomplished through HPLC leading to the identification of phenolic acids (chlorogenic, caffeic, and Ferulic acids), flavonoids (quercetin, apigenin, and kaempferol) with quercetin, apigenin, and chlorogenic as dominant constituents. Molecular docking study was performed to corroborate the in vitro results. Cytotoxicity testing on normal human skin fibroblasts indicated that the methanolic extract of the fifth nymphal stage was nontoxic up to 1000 µg/mL. No toxicity or mortality was observed at doses up to 4000 mg/kg.bw in zebrafish and 1500 mg/kg in mice. These findings confirm the antioxidant potential of *S. gregaria* nymphal extract with no in vitro or in vivo toxicity.

## Graphical abstract

The edible insect *Schistocerca gregaria* exhibits potent antioxidant activity and represents a sustainable food source while contributing to pest management. Integrated in vitro antioxidant assays, molecular docking with heme oxygenase, cytotoxicity evaluation in human skin fibroblasts, and in vivo safety studies via different administration routes demonstrate its promising safety profile. Further studies on chronic exposure are warranted.

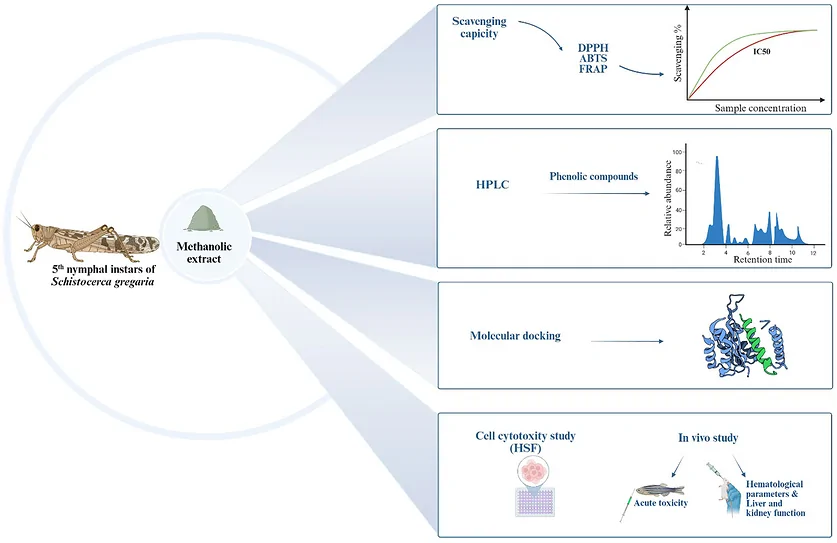

## 1 Introduction

Insects represent over 60% of all biodiversity on planet [1]. Entomophagy, or the eating of insects and invertebrates, has long been part of human history, serving religious, cultural, and nutritional roles, especially in tropical and subtropical regions among rural communities in Africa and Asia [2, 3, 4]. Recently, interest in edible insects has grown due to their bioactive compounds such as phytosterols, tocopherols, and phenolic compounds [5].

The urgent need to reduce the ecological impact of food production has increased global interest in edible insects as having a negligible ecological footprints, water and greenhouse gas (GHG) emission, which enable them to fulfill the population demand for alternative sustainable protein source [4, 6, 7, 8, 9]. Compared to conventional livestock and larger animals, edible insects require less rearing land or less breeding space [10]. They have been associated with consuming organic waste as food [9].

Since the (FAO) report titled “Edible Insects: Future Perspectives of Food and Nutrition Security”, insect ingestion is now a new trend in food research and science especially since 2013 [11].

In general, the medicinal uses of insects include the tumor growth suppression, antioxidant, antibacterial, anti‐inflammatory, and immunomodulation properties as well as the control of intestinal bacterial flora, reduction of blood pressure, regulation of lipid and blood pressure, and cardiovascular protection [12, 13]. In mealworms, housefly maggots and crickets, content of unsaturated fatty acids can help to reduce cardiovascular disease developing risks [14]. Nutritional composition of insects relies on different factors including the species, the diet, the developmental stage, and other factors [7].

Beetles were the most widely consumed insect species accounting for 31% then caterpillars (18%); then bees, ants, and wasps (14%); then crickets, locusts, and grasshoppers (13%); then true bugs and scale insects (10%) with smaller contributions from dragonflies and termites (3% each) and flies (2%) [9]. Edible insects such as crickets, grasshoppers, silkworms, and locusts are notably rich in fiber, essential amino acids, omega‐3 fatty acids, and antioxidant and anti‐inflammatory compounds, frequently outperforming orange juice in nutritional value [6, 13, 15, 16]. The consumption of insects has a positive impact on the way antioxidant enzymes regulated and exhibit excellent antioxidant properties [6, 17]. Several studies testing the antioxidant impact of edible insects using animals [6], cell cultures, or in vitro experiments [18] and showed positive results.

Field crickets (*Gryllus bimaculatus*), bamboo caterpillars (*Omphisa fuscidentalis*) and frozen silkworms (*Bombyx mori*) have antioxidant activity [19, 20]. Son et al. [21] reported that water‐soluble extract from *Tenebrio molitor* was effective antioxidant using DPPH, ABTS, and FRAP antioxidant method. According to Zielińska et al. [16], protein hydrolysates extracts of *S. gregaria*, *T. molitor*, and *Gryllodes sigillatus* showed positive results with DPPH and ABTS scavenging capacity method and Fe^2+^ chelating activity. Moreover, an antioxidant capacity was investigated using DPPH, FRAP, and ABTS activity method on water‐soluble extract (500 µg/mL) of *Apis mellifera* and *G. bimaculatus*, and showed positive results [22]. *Vespa affinis* L. aqueous extract (1.25–10 µg/µL) can increase the activity of glutathione s‐transferase (GST) and catalase (CAT) endogenous antioxidant enzymes in vitro studies, while ABTS was utilized in protein hydrolysates of *Hermetia illucens* (2 mg/mL) and recorded a substantial antioxidant activity [23]. The desert locust, *S. gregaria* (Orthoptera: Acrididae), is a major agricultural pest affecting a wide range of crops, particularly cereals, across Africa and Asia, with severe outbreaks reported in countries such as Eritrea, Ethiopia, Somalia, Yemen, and Saudi Arabia [24]. To date, many research on the desert locust has focused on its nutrition, physiology, control, metabolism, and biochemistry [24, 25]. Recently, research on *S. gregaria* has increasingly focused on its potential as a source of nutritionally and therapeutically valuable compounds that could contribute to food security in Africa [24]. As a result, locust control efforts could include rearing and consumption as part of the integrated management.

The current research aimed to observe the nutraceutical and pharmaceutical values of the methanolic extract of *S. gregaria* by investigating its biological activities as antioxidant agent, characterizing its phenolic profile using HPLC, supporting by molecular docking modeling of the higher concentration compounds, assessing of cytotoxicity in vitro, and conducting biological safety tests through in vivo acute toxicity using Zebra fish and male Albino Swiss mice as the experimental models.

## 2 Materials and Methods

### 2.1 Insect Rearing

For colony establishment, *S. gregaria* (desert locust) adults were obtained from Section of Locust Research, Research Institute of Plant Protection, Dokki, Giza, Egypt. Fifth nymphal instars were used for experiments. In the laboratory, desert locust cages were kept at a temperature of 32 ± 2°C and relative humidity (RH) of (30%–50%). One hundred desert locusts were reared in wooden framed rearing cages (50 cm × 50 cm × 60 cm) covered with wire gauze in four sides, glass in the top and front side small door where daily routine feeding and cleaning were performed. Each rearing cage was supported with an electric bulb (100 watt) for heating and lighting. About 20 cm of sand layer in each cage bottom for egg laying and hatching. Water containers were placed under every cage leg and grease was applied to the electric wires to prevent ants from attacking the colony. *Trifolium alexandrinum* (Breseem) and mixture of 5% yeast powder and dry wheat bran in petri dishes were introduced for nymphs and adult feeding. Fifth nymphal instars were separated and transferred to another rearing cages for progeny rearing and experiments. Routine work includes removing the previous uneaten food, dead nymphs, faces, and counting the living insects was executed daily before introducing the fresh food and the cages were cleaned at regular intervals to avoid contamination and to keep locusts at outmost hygienic conditions [26, 27, 28].

### 2.2 Preparation of *S. gregaria* Extract

Briefly, twelve insect nymphs (total weight: 9.6 g) were homogenized and extracted using methanol for the second, third, fourth, and fifth nymphal instar of *S. gregaria* at a solvent‐to‐sample ratio of approximately 2:1 (v/w). Whole nymphs were used for extraction. The methanolic extract was prepared by grinding insects in a mortar followed by sonication for 20 min using ultrasonic microtip probe of 400 watt (ULTRASONIC Get 750, USA) and then filtered through filter paper Whattman No. 5. The supernatant was collected, and the pellet was re‐extracted three times. The combined supernatants were evaporated at 40°C, and the dried extract was stored at −20°C until use [29].The extraction yielded 430 mg of crude extract, corresponding to an extraction yield of 4.48%

### 2.3 Determination of Antioxidant Activity In Vitro

#### 2.3.1 DPPH Assay

Antioxidant activity of the extract was assessed at the Regional Center for Mycology and Biotechnology (RCMB), Al‐Azhar University.

The antioxidant activity of the test compound was assessed using the 2,2‐diphenyl‐1‐picrylhydrazyl (DPPH) free radical scavenging assay, as described by [30], with slight modifications. Freshly prepared (0.004% w/v) methanol solution of DPPH radical was prepared and stored at 10°C in the dark. A methanol solution of the extract with concentrations (2.5, 5, 10, 20, 40, 80, 160, 320, 640, and 1280 µg/mL) was prepared. A 40 µL methanolic sample was mixed with 3 mL DPPH solution, and absorbance was measured at 515 nm. Ascorbic acid served as control. All assays were performed in triplicate.

#### 2.3.2 ABTS Radical Scavenging Assay

The method was performed according to the procedure previously described [31, 32]. ABTS^+^ was generated by reacting 1.8 mM ABTS with 0.63 mM potassium persulfate in the dark for 16 h, then diluted with ethanol to an absorbance of 0.700 ± 0.030 at 734 nm. Measurements were conducted at room temperature, and sample concentrations were prepared in 80% methanol.

Subsequently, 190 µL of the radical solution was combined with 10 µL of diluted extract in a microtiter plate, and absorbance at 734 nm was recorded at 1‐min intervals for 13 min. All assays were conducted in triplicate [33].

#### 2.3.3 Ferric Ion Reducing Antioxidant Power Assay (FRAP)

The ferric reducing antioxidant power (FRAP) assay was evaluated according to the method described by [34], with slight modifications. The methanolic sample (1 mL) was mixed with sodium phosphate buffer (0.2 M, pH 6.6) and 1% potassium ferricyanide, incubated at 50°C for 20 min, then treated with 10% trichloroacetic acid and centrifuged at 1000 ×g for 10 min.

Briefly, 2.5 mL of the supernatant was mixed with 2.5 mL of deionized water and 0.5 mL of 0.1% ferric chloride, and absorbance was measured at 700 nm using a UV–vis spectrophotometer (Milton Roy, Spectronic 1201), with higher absorbance indicating greater reducing power.

#### 2.3.4 Total Antioxidant Capacity (TAC)

The total antioxidant capacity (TAC) was investigated by means of the method proposed by [35]. Absorbance was measured at 695 nm, and total antioxidant capacity was expressed as mg gallic acid equivalents per gram of extract.

### 2.4 Phenolic Profile Determination Using HPLC

The phenolic profile of the methanolic extract of *S. gregaria* was analyzed by HPLC using an Agilent 1260 system with a diode‐array detector and Eclipse XDB‐C18 column. The mobile phase consisted of water (A) and 0.05% trifuoroacetic acid in acetonitrile (B) at a flow rate 1 mL/min. The mobile was programmed consecutively in a linear gradient as follows: 0 min (82% A); 0–5 min (80% A); 5–8 min (60% A); 8–12 min (60% A); 12–15 min (82% A), and 15–16 min (82% A). The multiwavelength was monitored simultaneously at 280, 320, and 360 nm. The injection volume was 25 µL for each sample solution. The column temperature was set during the separation process to 35°C.

### 2.5 Molecular Docking

Molecular docking was performed to evaluate the interactions of the major HPLC‐identified compounds with human heme oxygenase‐1 (HO‐1; PDB ID: 1N3U) using AutoDock Vina v1.2 [36]. HO‐1 was selected because of its central role in cellular antioxidant defense through the Nrf2/HO‐1 pathway [37]. The HO‐1 crystal structure was obtained from the Protein Data Bank and prepared by removing the cocrystallized ligand, water molecules, and nonessential heteroatoms, followed by the addition of polar hydrogens and Kollman charges before conversion to PDBQT format. The eleven HPLC‐identified phytochemicals were prepared as flexible ligands with Gasteiger charges and converted to PDBQT format [37, 38]. Docking was performed using a grid box centered at *x* = 24.987 Å, *y* = 17.807 Å, and *z* = −25.756 Å (40 × 40 × 40 Å^3^), with an exhaustiveness of 8 and an energy range of 4 kcal mol^−^^1^. The lowest‐energy binding pose for each ligand was selected, and protein–ligand interactions, including hydrogen bonds, hydrophobic, π, electrostatic, and van der Waals interactions, were analyzed using BIOVIA Discovery Studio Visualizer.

### 2.6 Bioassay

#### 2.6.1 In Vitro Study

##### 2.6.1.1 Cell Culture

The Human Skin Fibroblast (HSF) cell line was purchased from Nawah Scientific Inc. (Mokatam, Cairo, Egypt). Cells were cultured in DMEM supplemented with 10% heat‐inactivated fetal bovine serum, 100 units/mL penicillin, and 100 mg/mL streptomycin, and maintained at 37°C in a humidified incubator with 5% CO_2_ [39].

##### 2.6.1.2 Cytotoxicity Assay

Cell viability was evaluated by the SRB assay. Cells (5 × 10^3^/well) were seeded in 96‐well plates, incubated for 24 h, then treated with the extract (0.01–100 µg/mL) for 72 h. Cells were fixed with 10% TCA, stained with 0.4% SRB, and the bound dye was dissolved in 10 mM Tris. Absorbance was measured at 540 nm [40].

#### 2.6.2 In Vivo Study

##### 2.6.2.1 Acute Toxicity on Zebrafish

Adult zebrafish (*Danio rerio*) of both sexes (60–90 days old), with comparable size (3.5 ± 0.5 cm) and weight (0.4 ± 0.1 g), were obtained from a local pet store. A total of 50 fish were acclimated in a 10‐L glass aquarium (30 × 15 × 20 cm) containing dechlorinated tap water, equipped with aeration and a submerged filter, at 25°C and pH 7.0 under a 14:10 h light/dark photoperiod. During the acclimation period, 20% of the tank water was replaced daily. The fish received feed ad libitum 24 h prior to the experiments, as 1 % of total fish weight. The wastes were removed daily. Zebrafish were acclimated in glass tanks with dechlorinated water and fasted for 24 h before and during experiments. Five fish per group were used, in accordance with Directive 2010/63/EU to minimize animal use. Experimental group sizes were based on previous studies. Extract doses were prepared in 3% DMSO, and individual zebrafish (*n* = 5 per dose) were intraperitoneally injected with 20 µL at 250, 500, 1000, 2000, or 4000 mg/g. Mortalities were monitored over 96 h, and the 96‐h median lethal concentration (LC_50_) was calculated from the daily death counts [41]. All experimental protocols were reviewed and approved by the institutional ethical committee for the use of animals in scientific research (MUFS/F/GE/825).

##### 2.6.2.2 Acute Oral Toxicity Study on Mouse Model

Thirty adult healthy male Albino Swiss mice weighing 29.53 ± 4.98 g (6–8 weeks old) were purchased from the animal facilities, an Egyptian Holding Company for Biological Products and Vaccines (VACSERA), Giza, Egypt. Mice were housed for 2 weeks to adapt to their new environment before the beginning of the experiment. Mice were randomly allocated into six groups (*n* = 5 per group). Prior to treatment, animals were fasted for 18 h while having free access to water. After that, they administered various ascending doses of methanolic extract dissolved in DMSO 5%. A single oral dose was administered by gavage according to body weight. Group I served as the normal control and received distilled water, group II received 5% DMSO, while groups III–VI were treated with the methanolic extract at doses of 250, 500, 1000, and 1500 mg/kg body weight, respectively. Close observation of the animals were maintained for the first 30 min., then for 1 and 3 h. For the first 4 h and then at every other day for a total of 14 days, each group was rigorously monitored for any harmful consequences. At the end of the study, mice were anesthetized by isoflurane, and then the blood was then taken from the orbital sinuses for haematological, liver, and kidney functions. An automatic hematology analyzer (NS Bio‐Tec Hema 21, Egypt) was utilized to determine the hematological assay. Serum samples were obtained by centrifuging blood at 3500 rpm for 10 min. Liver and kidney function parameters were subsequently analyzed using commercially available BioMed kits [42]. The protocol of the study was approved according to the Institutional Animal Ethical Committee of the Faculty of Science, Menoufia University (Approval No., MUFS/F/GE/8/25).

### 2.7 Statistical Analysis

Data are presented as mean ± SD. Cell viability percentages were determined by regression analysis, and IC_50_ values for DPPH and ABTS and EC50 for FRAP were calculated using a nonlinear curve‐fitting model. The data was analyzed using graph padprism 8, San Diego, CA, USA. Extra‐ sum‐ of‐ squares F test together with Bonferroni correction was used to compare between various age stages and ascorbic acid, and between fifth instar and other age stages. Confidence interval was provided to indicate no overlap between ages as indication of significance. The statistical significance of differences between different groups in biological experiments was assessed using ANOVA test. Post hoc multiple comparison was conducted using tukey test. A value of *p* ˂ 0.05 denoted cutoff of statistical significance.

## 3 Results

### 3.1 Antioxidant Activity of *S. gregaria* Extract

**FIGURE 1:**
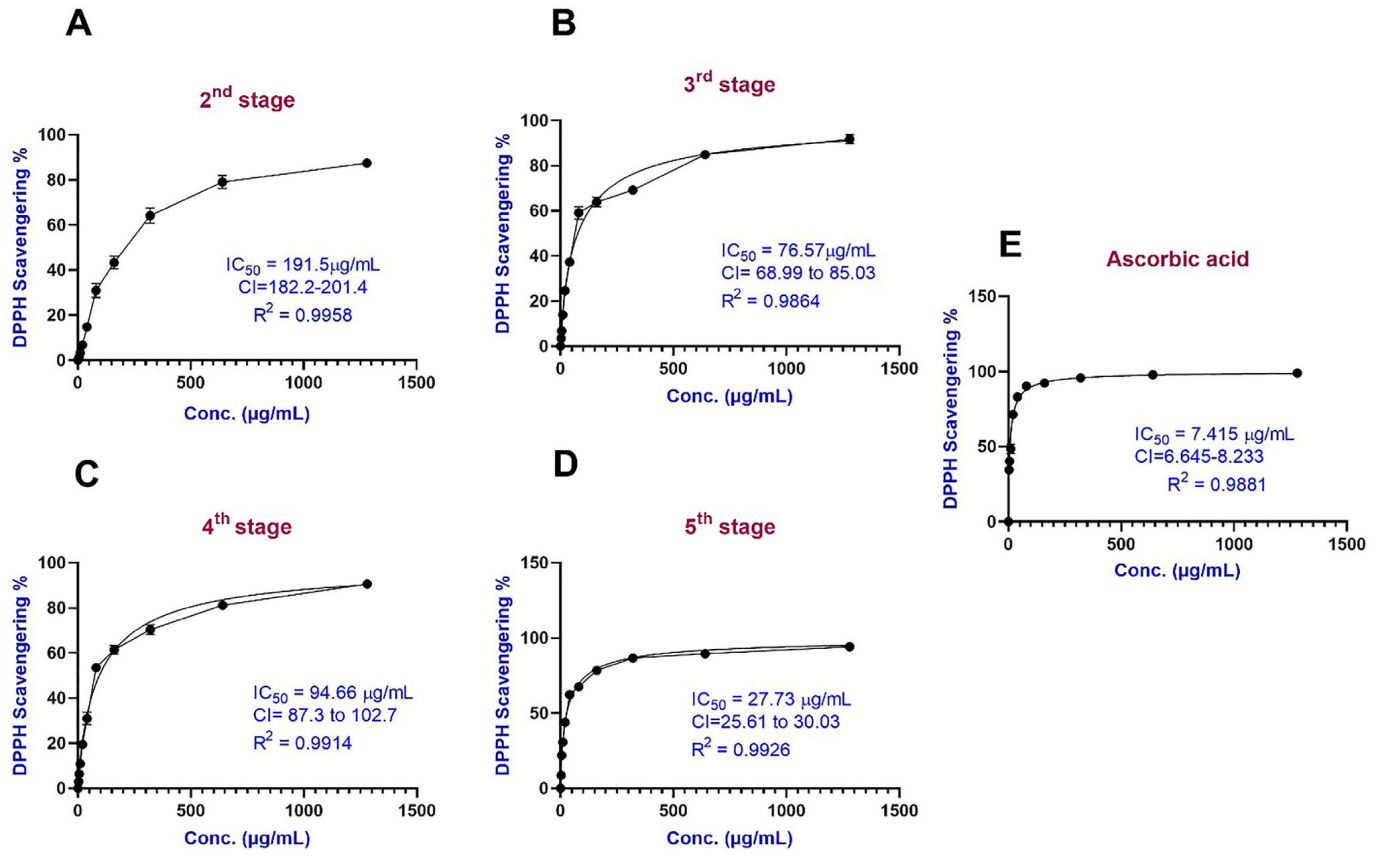
DPPH scavenging assay of *S. gregaria* nymphal instars. (A) Second: second nymphal instar, (B) Third: third nymphal instar, (C) Fourth: fourth nymphal instar, (D) Fifth: fifth nymphal instar, (E) Ascobic acid. Data are expressed as mean ±SD, (*n* = 3).

To confirm the in vitro antioxidant properties of the nymphal extract, DPPH, ABTS, and FRAP assay were performed. The results in Figure 1 showed different *S. gregaria* nymphal stages methanolic extracts’ DPPH radical scavenging activity.

The DPPH free radical scavenging activity of the extracts increased with increasing concentration of *S. gregaria* nymphal instars (2.5 to 1280 µg/mL). For the second instar extract, the scavenging activity ranged from 0.74% to 87.49%, with an IC_50_ of 191.5 µg/mL. The third instar extract showed activity ranging from 3.46% to 91.76% at the same concentrations, with a lower IC_50_ of 76.57 µg/mL. The fourth instar extract demonstrated scavenging activity from 2.97% to 90.62%, with an IC_50_ of 94.66 µg/mL, while the fifth instar extract showed the highest activity, ranging from 8.73% to 94.23% over the same concentration range, with an IC_50_ of 27.73 µg/mL. Ascorbic acid, used as the reference standard, exhibited an IC_50_ of 7.415 µg/mL, as represented in Figure 1.

**FIGURE 2:**
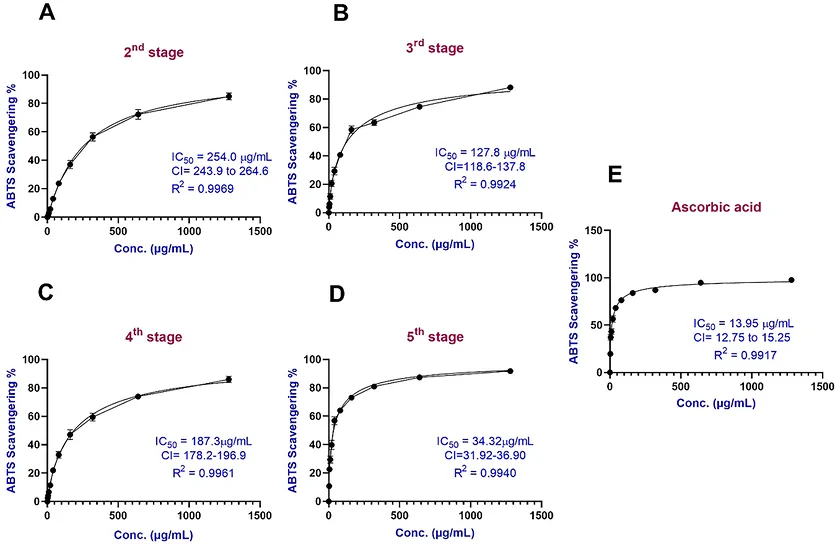
ABTS scavenging assay of *S. gregaria* nymphal instars. (A) Second: second nymphal instar, (B) Third: third nymphal instar, (C) Fourth: fourth nymphal instar, (D) Fifth: fifth nymphal instar, (E) Ascorbic acid. Data are expressed as mean ±SD, (*n* = 3).

The results in Figure 2 showed different *S. gregaria* nymphal stages methanolic extracts’ ABTS radical scavenging activity. The ABTS scavenging free radical percent ranged from 0.67% to 84.95% with IC_50_ 254.0 µg/mL in the second nymphal instar methanolic extract, ranged from 3.95% to 88.21% with IC_50_ 127.8 µg/mL for third nymph extract, where the ABTS scavenging free radical percent of fourth and fifth nymphal instar methanolic extracts ranged from 2.53% to 86.07% with IC_50_ 187.3 µg/mL and from 10.75% to 91.75% with IC_50_ 34.32 µg/mL, respectively. Ascorbic acid, used as the reference standard, exhibited an IC_50_ of 13.95 µg/mL.

**FIGURE 3:**
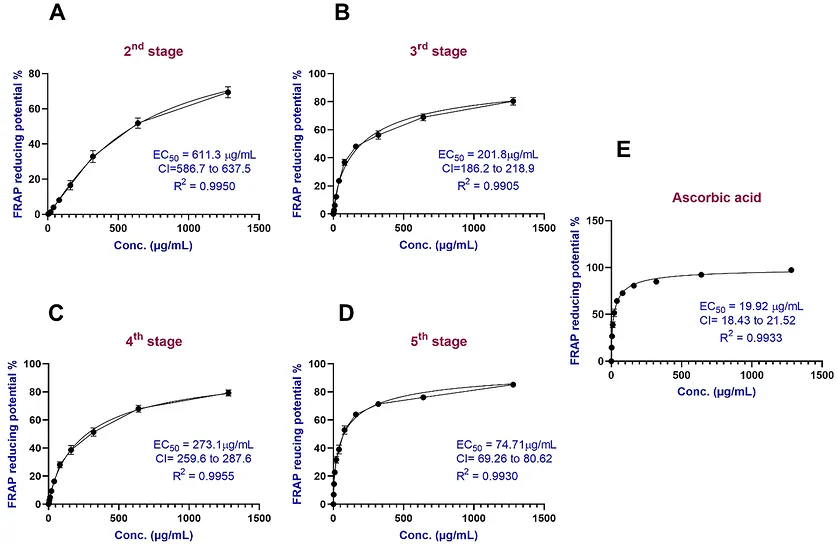
FRAP reducing potential of *S. gregaria* nymphal instars. (A) Second: second nymphal instar, (B) Third: third nymphal instar, (C) Fourth: fourth nymphal instar, (D) Fifth: fifth nymphal instar, (E) Ascorbic acid. Data are expressed as mean ±SD, (*n* = 3).

The results in Figure 3 showed the FRAP reducing potential of different *S. gregaria* nymphal stage methanolic extracts. The FRAP reducing activity ranged from 0.23% to 69.43% with EC_50_ 611.3 µg/mL in the second nymphal instar methanolic extract, ranged from 1.29% to 80.35% with EC_50_ 201.8 µg/mL for third nymph extract. The FRAP scavenging activity of fourth and fifth nymphal instar methanolic extracts ranged from 0.94% to 79.31% with EC_50_ 273.1 µg/mL and from 6.79% to 85.23% with EC_50_ 74.71 µg/mL, respectively. This suggests that the methanolic fifth nymphal extract had better reducing potential than the methanolic extract of second, third, and fourth nymph instars. Ascorbic acid, used as the reference standard, exhibited an EC_50_ of 19.92 µg/mL. Across all three antioxidant assays, extra sum‐of‐squares F‐test comparisons showed that the inhibitory/ effective concentrations (IC_50_ for DPPH and ABTS; EC_50_ for FRAP) differed significantly among the nymphal instars, with the fifth instar consistently showing the lowest (most potent) IC_50_/EC_50_ across all three assays. The fifth instar showed the smallest fold‐difference from the reference standard across all assays, indicating relatively stronger antioxidant potential compared to the earlier developmental stages (Table S1), for detailed statistics.

### 3.2 Total Antioxidant Capacity (TAC)

The provided samples had different antioxidant activity which expressed as milligrams Gallic acid Equivalent (GAE) per gram sample. Total antioxidant capacity of *S. gregaria fifth* nymphal stage methanolic extracts were determined using ascorbic acid as a reference. Methanolic extract of fifth nymphal instar exhibited a lesser antioxidant capacity than ascorbic acid, with TAC value of 17.23 ± 3 mg GAE/g compared to 69.84 ± 4 mg GAE/g for ascorbic acid.

### 3.3 Phenolic Compounds Profile of *S. gregaria* Methanolic Extract

**FIGURE 4:**
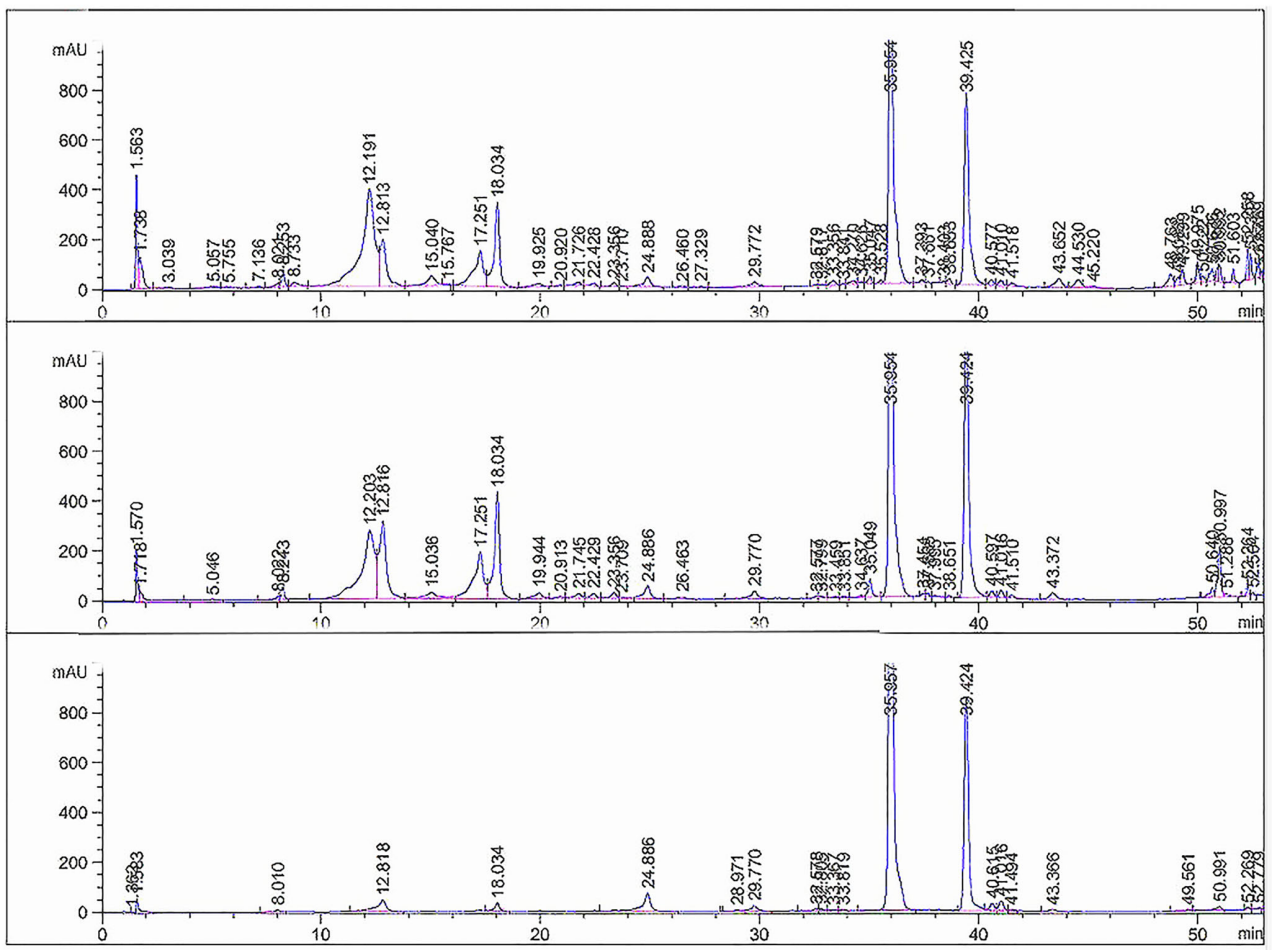
HPLC chromatogram of methanolic extract of fifth nymphal instar of *S. gregaria*.

The phenolic compounds profile of *S. gregaria* fifth nymphal instar methanolic extract was analyzed colorimetrically using HPLC (Table 1 and Figure 4). A total of eleven polyphenolic compounds were identified, with Quercetin being the most abundant. Ten phenolic acids were detected, among which Quercetin (682.33 µg/g extract), Apigenin (300.78 µg/g), and Chlorogenic acid (206.63 µg/g) were the predominant components. Additional phenolic acids identified included Caffeic acid (72.16 µg/g). Our results showed that sinapic was the minor compound among the phenolic compounds mentioned.

**TABLE 1:** Phenolic profile of desert locust *S. gregaria* methanolic extract using HPLC.

Compound	RT	Peak area	Concentration (µg/g)
**Qurecetin**	36.3	4.59E + 04	682.327
**Apigenin**	39.2	1.62E + 04	300.7775
**Chlorogenic**	12.7	1.17E + 04	206.6304
**Caffeic**	13.5	7438.83	72.16295
**Vanillic**	16	1206.87	14.07986
***p*‐coumaric**	25.4	937.3375	10.95801
**Kaempferol**	40.8	434.5522	7.290293
**Chrysin**	51.5	494.7697	6.655499
**Ferulic**	20.6	588.2609	5.674708
**Cinnamic**	35.1	267.377	1.710151
**Sinapic**	21.5	103.1599	1.072673

RT: Retention time (min)

### 3.4 Molecular Docking

**FIGURE 5:**
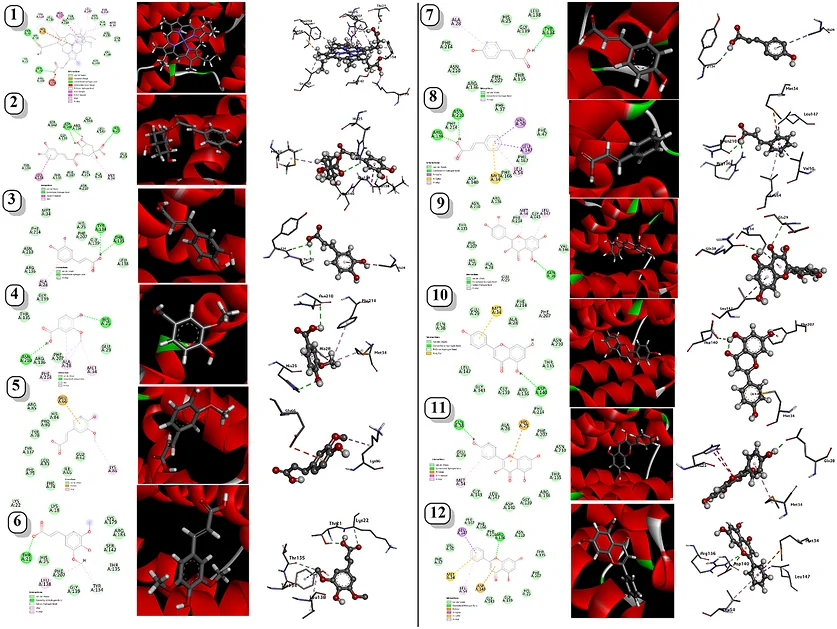
Representative two‐dimensional (2D) protein–ligand interaction diagrams and three‐dimensional (3D) binding poses of the HPLC‐identified phytochemicals within the active site of human heme oxygenase‐1 (HO‐1; PDB ID: 1N3U). (1) Original cocrystallized ligand; (2) apigenin; (3) caffeic acid; (4) chlorogenic acid; (5) chrysin; (6) cinnamic acid; (7) *p*‐Coumaric acid; (8) vanillic acid; (9) kaempferol; (10) quercetin; (11) sinapic acid; and (12) ferulic acid.

Molecular docking was performed to evaluate the binding of the major HPLC‐identified compounds from the methanolic extract of the fifth nymphal instar of *S. gregaria* to human heme oxygenase‐1 (HO‐1; PDB ID: 1N3U). Binding affinities and key protein–ligand interactions are presented in Table 1, and representative binding modes are shown in Figure 5.

All compounds were successfully accommodated within the HO‐1 active site, with binding energies ranging from −5.2 to −7.8 kcal mol^−^^1^, compared with −9.8 kcal mol^−^^1^ for the re‐docked cocrystallized ligand. Quercetin exhibited the strongest predicted binding (−7.8 kcal mol^−^^1^), followed by apigenin (−7.7), chrysin (−7.6), kaempferol (−7.5), and chlorogenic acid (−7.4 kcal mol^−^^1^). Vanillic acid showed the weakest affinity (−5.2 kcal mol^−^^1^), whereas the remaining phenolic acids displayed intermediate values (−5.6 to −5.9 kcal mol^−^^1^).

Binding was primarily stabilized by hydrogen bonds, together with carbon–hydrogen bonds, π‐interactions, electrostatic interactions, and van der Waals contacts (Table 2). Flavonoids formed more extensive interaction networks than phenolic acids, reflecting their multiple hydroxyl groups and aromatic rings, which enhance hydrogen‐bonding and π‐mediated interactions. Although phenolic acids established fewer contacts, all compounds occupied the same HO‐1 binding pocket with similar orientations, and differences in interaction patterns accounted for the observed variation in binding affinity.

**TABLE 2:** Molecular docking scores and key interactions of HPLC‐identified compounds with (PDB ID: 1N3U).

Compound	S (Kcal/mol) interaction	Key amino acid	Type of interaction	Distance Å
Original ligand	−9.8	THR 21	Conventional H‐bond	4.29
		SER 142	Conventional H‐bond	4.80
		HIS 25	Attractive charge π‐donor H‐bond	3.48
Chlorogenic acid	−7.4	HIS 25	Conventional H‐bond	4.64
		ASP 140	Conventional H‐bond	2.98
Caffeic	−5.9	TYR 134	Conventional H‐bond	4.39
		THR 135	Conventional H‐bond	4.28
Vanillic	−5.2	HIS 25	Conventional H‐bond	4.98
		ASN 210	Conventional H‐bond	4.46
		MET 34	π‐alkyl	4.93
Ferulic	−5.7	GLU 66	π‐anion	5.4
		LYS 86	Alkyl	3.94
Sinapic	−5.6	THR 21	Conventional H‐bond	3.70
		TYR 134	Carbon H‐bond	4.15
		LYS 22	Carbon H‐bond	3.78
*p*‐Coumaric	−5.8	TYR 134	Conventional H‐bond	3.21
		ALA 28	π‐alkyl	7.11
Cinnamic	−5.6	ASN 210	Conventional H‐bond	4.0
		ARG 136	Conventional H‐bond	5.36
		VAL 50	π‐σ	5.71
		MET 34	π‐sulfur	6.63
Quercetin	−7.8	GLN 38	Conventional H‐bond	4.29
		GLU 29	Carbon H‐bond	3.57
		LEU 147	π‐alkyl	5.67
Apigenin	−7.7	ASP 140	Conventional H‐bond	3.42
		MET 34	π‐sulfur	5.32
		PHE 207	Carbon H‐bond	2.80
Kaempferol	−7.5	GLN 38	Conventional H‐bond	3.92
		HIS 25	π‐cation	6.12
		MET 34	π‐alkyl	6.12
Chrysin	−7.6	ARG 136	Conventional H‐bond	5.87
		ASP 140	π‐anion	5.15
		MET 34	π‐sulfur	6.9
		LEU 147	π‐σ	5.6

### 3.5 Cell Cytotoxicity Study

**FIGURE 6:**
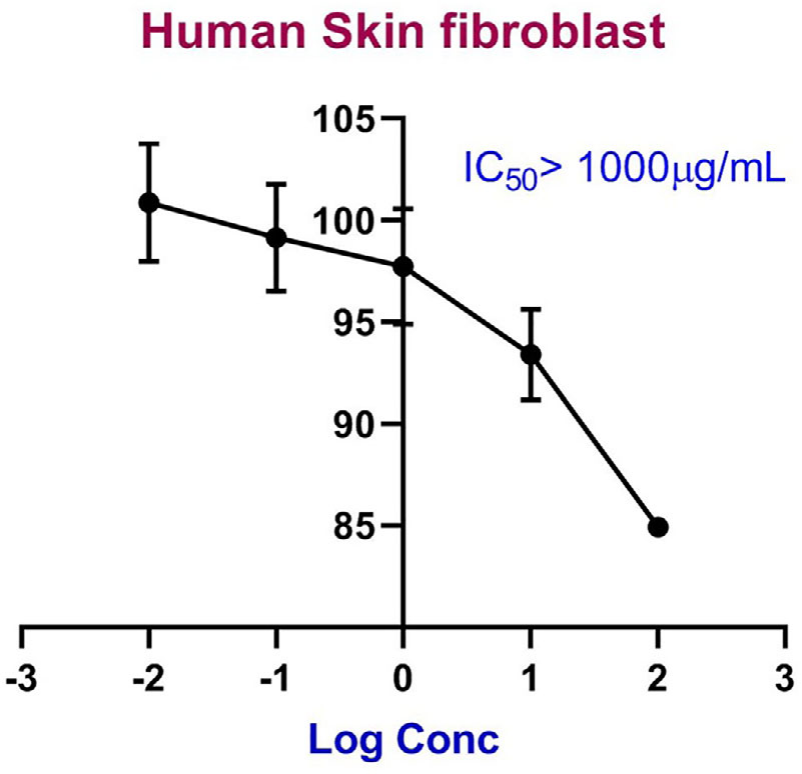
Cytotoxicity analysis of the methanolic fifth nymphal extract using the SRB assay. Dose–response curve shows the effect of the extract on HSF viability and the obtained IC_50_ after 72 h. Data are expressed as mean ± SD, *n* = 3.

The cytotoxicity effect of *S. gregaria* methanolic extract on HSF was evaluated by SRB assay. The cell viability at concentrations (0.01, 0.1, 1, 10, and 100 µg/mL) of the extract was measured to be 100.89 ± 2.91%, 99.15 ± 2.64%, 97.74 ± 2.82%, 93.41 ± 2.23%, and 84.94 ± 0.37% in HSF cells, respectively. The methanol extract of *S. gregaria* fifth nymphal instar exhibited IC_50_ value of about 1000 µg/mL, indicating a very week toxic effect on normal cells. Figure 6.

### 3.6 Acute Toxicity on Adult Zebrafish

*S. gregaria* methanolic extract (250 to 4000 mg/Kg.bw; 20 µL; i.p.) caused no mortality in adult zebrafish. Moreover, up to 4000 mg/Kg.bw, no morphological or behavioral abnormalities were recorded.

### 3.7 Acute Toxicity on Adult Male Albino Mice

Up to 14 days of administration, no death or adverse clinical signs were observed in male mice who received 250, 500, 1000, and 1500 mg/kg of fifth *S. gregaria* nymphal methanolic extract.

### 3.8 Biomarkers Assessments

#### 3.8.1 Hematological Parameters

**FIGURE 7:**
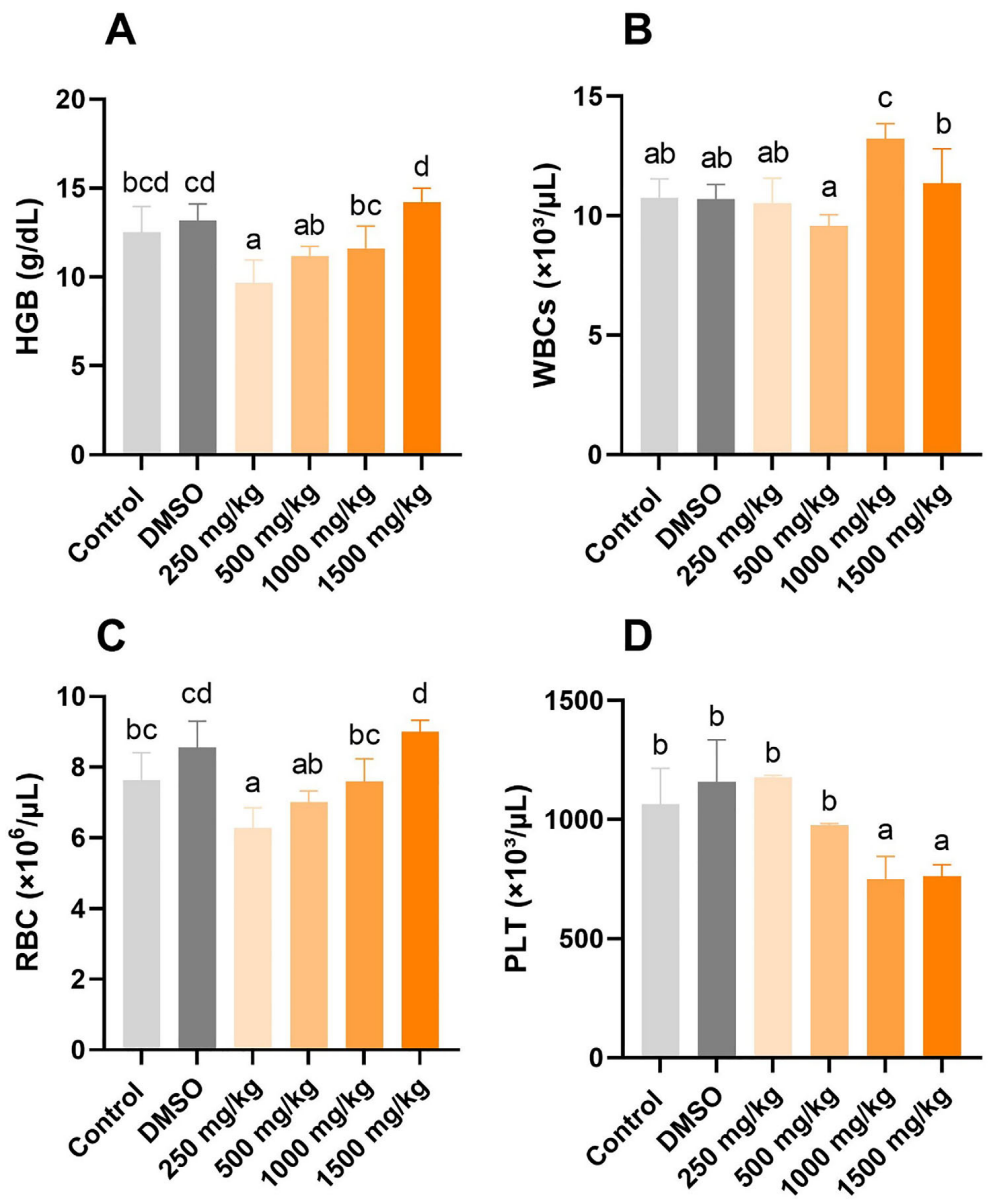
Effect of nymphal methanolic extract on the haematological parameters of adult male Swiss albino mice. Results were depicted as mean ± standard deviation (*n* = 5), (A) hemoglobin HGB, (B) white blood cells WBCs, (C) red blood cell RBCs, (D) platelet PLT, with different letters above columns indicate significance difference between groups *p* < 0.05.

A significant decrease in hemoglobin (HGB) levels was observed in the group treated with 250 mg/kg (9.67 ± 1.29 g/dL) compared with the control group (12.50 ± 1.47 g/dL), Figure 7.

A significant increase (13.22 ± 0.64) was recorded with 1000 mg/kg treated group compared with the control (10.75 ± 0.79 ×10^3^/µL), Figure 7.

The group treated with 1500 mg/kg showed a significant increase (9 ± 0.33 ×10^6^/µL) in RBC count compared with the control group (7.63 ± 0.79 ×10^6^/µL). Also, there was a significant decrease in group treated with 250 mg/kg (6.28 ± 0.58 ×10^6^/µL), Figure 7.

However, Platelet (PLT) levels were significantly decreased in groups treated with 1000 and 1500 mg/kg (750.20 ± 95.06 and 763 ± 47.31 ×10^3^/µL, respectively) compared with the control (1066.2 ± 148.9 ×10^3^/µL), Figure 7.

#### 3.8.2 Liver and Kidney Functions

**FIGURE 8:**
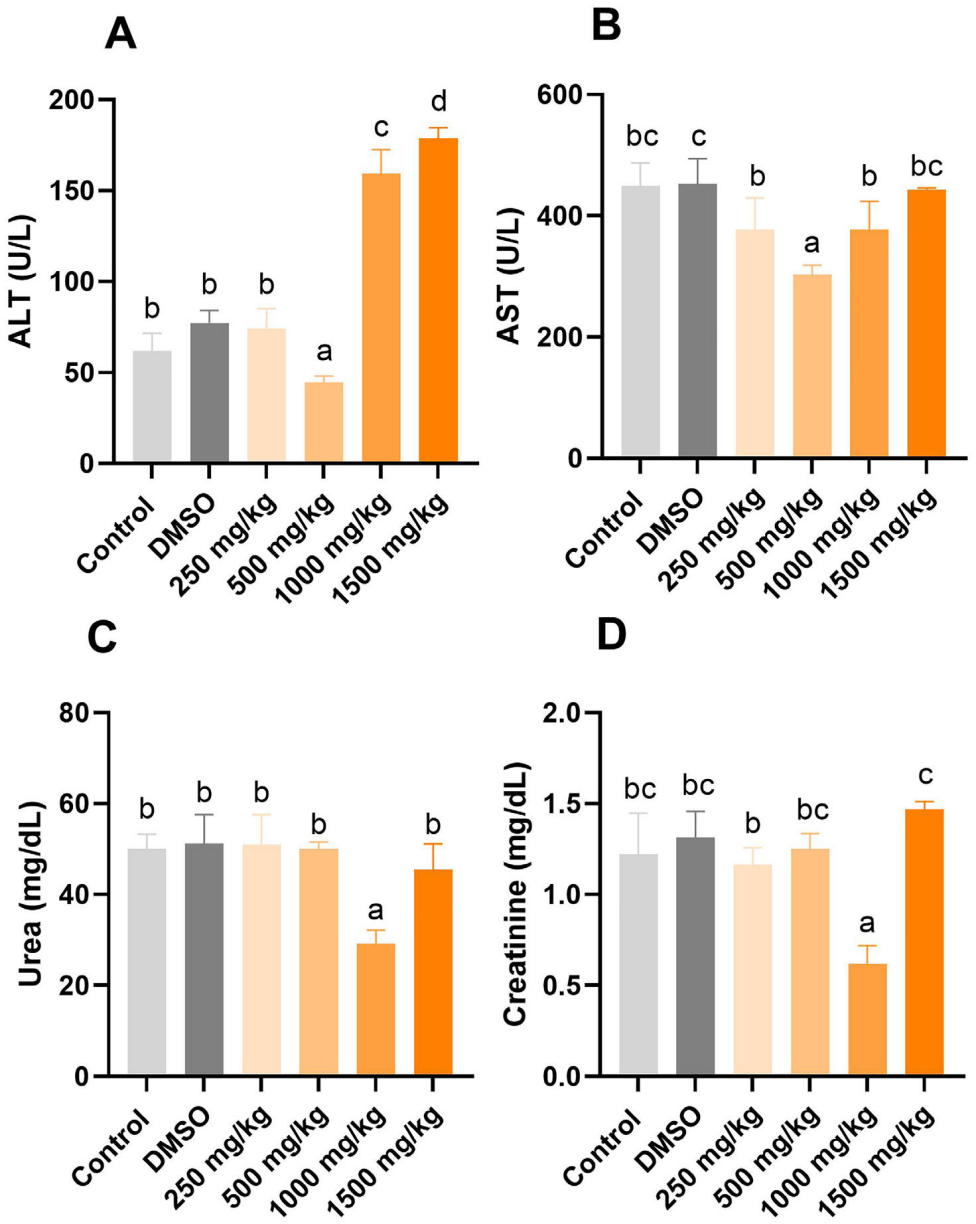
Effect of nymphal methanolic extract on the liver and kidney functions of adult male Swiss albino mice. Results were depicted as mean ± standard deviation (*n* = 5), (A) alanine aminotransferase ALT, (B) aspartate aminotransferase AST, (C) urea, (D) creatinine, with different letters above columns indicate significance difference between groups *p* < 0.05.

Treatment of male mice with nymphal methanolic extract at doses of 1000, and 1500 mg/kg, resulted in a significant elevation in alanine aminotransferase (ALT) levels (159.20 ± 13.44, and 178.75 ± 5.87 U/L, respectively) compared with the control group (62.15 ± 9.51 U/L). Meanwhile, treatment with 500 mg/kg of nymphal methanolic extract induced a significant decline in ALT (44.5 ± 3.54 U/L) compared with the control group, Figure 8.

Meanwhile, treatment with 500 mg/kg of nymphal methanolic extract induced a significant decline in aspartate aminotransferase (AST) levels (303 ± 15 U/L) compared with the control group (448.80 ± 38.46 U/L), Figure 8.

A significant decrease in Urea levels was observed in mice treated with 1000 mg/kg of the extract (29.25 ± 2.95 mg/dL) compared with the control group (50 ± 3.32 mg/dL), Figure 8.

A significant reduction in creatinine levels (0.62 ± 0.098 mg/dL) was observed in mice treated with 1000 mg/kg of the extract, compared with the control group (1.22 ±.23 mg/dL), Figure 8.

## 4 Discussion

Edible insects, particularly crickets, grasshoppers, silkworms, and locusts, are rich in bioactive compounds that show strong antioxidant and anti‐inflammatory activities, including phenolic compounds [5, 6, 15, 16]. Phenolic compounds are considered primary antioxidants as they can neutralize free radicals, quench singlet and triplet oxygen species or decompose peroxide species [43]. *S. gregaria* consume phenolic compounds with plant‐based diet, and metabolize them into new derivatives with potential health benefits for humans [24]. The high levels of phenolics and flavonoids present in edible insects can enhance direct ROS scavenging, helping reduce oxidative stress linked to inflammation and diseases such as cardiovascular disorders, cancer, diabetes, and Alzheimer's disease [44, 45, 46].

In this study, data cleared that the extract showed good antioxidant activity. Our study is in agreement with Mariod, [47] who reported that the dry matter of *Locusta migratoria* has antioxidant activity against oxidative stress through the capacity to chelate metal ions. Our findings aligned with several studies reporting antioxidant activity in edible insects such as in *B. mori* pupa hydrolysate [48], mealworm *T. molitor* [49], methanolic extracts of *Serrognathus platymelus* [50], adult *Holotrichia parallela* extracts [51], the injected *Musca domestica* larval hemolymph [52], and *Protaetia brevitarsis* at different life stages [53], as well as *V. affinis* extracts [18]. Protein hydrolysates of *H. illucens* larvae also showed strong DPPH, hydroxyl radical scavenging, and metal‐chelating activities [54]. FRAP assay based in single electron transfer and ORAC (oxygen radical absorbance capacity) assay based in hydrogen atom transfer were measured in adult crickets *Acheta domesticus* aqueous extract [55].

In this work, HPLC analysis of *S. gregaria* methanolic extract revealed eleven phenolic compounds, the major phenolic compounds obtained were Quercetin, Apigenin, and Chlorogenic acid. Quercetin have varied bio‐functions, including anti‐cancer, anti‐inflammatory, and renal‐protective effect [56]. P‐coumaric and caffeic acid, have antimicrobial, antioxidant, and anti‐inflammatory properties, and may decrease the negative impact of oxidative stress‐related diseases [56]. Several researches focused on certain phenolic compounds that present in insect cuticle, such as quercetin, ferulic acid, and catechin [51, 57]. The aqueous and alcoholic extracts of *Vespa orientalis* larvae have amounts of phenolic compounds, including vanillic acid, caffeic acid, and ferulic acid [58]. Moreover, quercetin was also found in silk worm *B. mori* extract [59].

The molecular docking analysis provided structural insight into the interactions between the HPLC‐identified compounds and human heme oxygenase‐1 (HO‐1), a key enzyme in the Nrf2/HO‐1 antioxidant defense pathway. Although none of the compounds achieved the binding affinity of the cocrystallized ligand, several showed favorable docking scores and stable binding within the HO‐1 active site. As molecular docking is a predictive computational approach, these findings provide mechanistic support for the experimentally observed antioxidant activity rather than direct evidence of HO‐1 activation [36, 60].

Among the tested compounds, the flavonoids quercetin, apigenin, chrysin, and kaempferol exhibited stronger binding affinities than the phenolic acids, consistent with previous studies attributing their enhanced protein binding to multiple hydroxyl groups and conjugated aromatic rings that facilitate hydrogen bonding and π‐mediated interactions [60, 61]. Hydrogen bonds were the predominant interactions, complemented by hydrophobic, π‐interactions, electrostatic, and van der Waals contacts. Residues including HIS25, GLN38, ASP140, MET34, and LEU147 were repeatedly involved in ligand recognition, highlighting their importance in HO‐1 binding [61].

Among the phenolic acids, chlorogenic acid displayed the strongest binding affinity, likely due to its multiple hydroxyl groups and ester functionality, whereas caffeic, ferulic, sinapic, *p*‐coumaric, cinnamic, and vanillic acids showed weaker docking scores. This agrees with reports describing chlorogenic acid as an effective antioxidant through stable hydrogen‐bonding interactions with proteins involved in oxidative stress regulation [62].

Notably, quercetin, apigenin, and chlorogenic acid were major constituents of the methanolic extract and also exhibited the most favorable HO‐1 binding profiles, providing a structural basis for the strong antioxidant activity observed in the DPPH, ABTS, FRAP, and total antioxidant capacity assays. Similar relationships between phytochemical composition, molecular docking, and antioxidant activity have been reported for *Philodendron heleniae* extracts and *Heterotrigona itama* propolis, supporting the biological relevance of the present findings [63, 64, 65].

Toxicological evaluations serve as a cornerstone in pharmacological research and pharmaceutical development, helping to establish a compound's safety profile before its clinical application in humans [66]. In vitro method is a valuable tool for assessing genotoxicity and comparing the potential health risks of edible insects. Turkez et al. [67] evaluated the genotoxic potentials of water soluble extracts of migratory locust (*L. migratoria*) on human blood cells, the results indicated that the extracts did not show any toxicity at tested concentrations from 0 to 1000 mg/L. Hence, this extract could be applied as adjuvants for anticancer medications, as well as antioxidants and anti‐inflammatory compounds. Zebrafish is a widely used vertebrate model in genetics, toxicology, development, and regeneration, favored for its small size, ease of husbandry, and high reproductive capacity [42]. In this study, zebra fish adults provide a rapid approach confirming the safety of *S. gregaria* nymph methanolic extract up to 4000 mg/Kg.bw. Further research on the methanolic extract of *S. gregaria* is needed to confirm the biochemical findings and ensure the extract's safety and mode of action.

Mouse models are commonly used to predict human responses due to their genetic and physiological similarities to humans [42]. Hematological and blood chemistry analyses are key indicators of physiological stress and potential tissue damage, offering important insights into the cellular integrity of internal organs [66]. This study was performed to demonstrate a No Observed Adverse Effect Level (NOAEL) for a methanolic extract of *S. gregaria* fifth nymph instar and the results clearly demonstrated that the extract was safe up to 1500 mg/kg in male mice. Hepatic and renal functions were evaluated using standard blood chemistry markers, including ALT, AST, urea, and creatinine. No signs of toxicity were detected, as urea and creatinine levels remained within normal ranges, AST showed no significant differences among groups, and ALT remained unchanged up to 500 mg/kg, indicating that *S. gregaria* methanolic extract does not cause hepatic or renal toxicity.

Yu et al. [68] investigated the effect of safety and toxicity of aqueous extract of *G. bimaculatus* in sprague‐dawley rat, the results demonstrated that 3000 mg/kg is a NOAEL dose for *G. bimaculatus* extracts in Sprague Dawley rats. *M. domestica* demonstrated trypanocidal activity against Trypanosoma brucei brucei–infected albino rats, accompanied by alterations in selected biochemical enzymes and hematological parameters [69]. Yunita, et al. (2026) [70] evaluated the acute oral toxicity and hepatic responses of ethanolic* Paederia foetida* leaf extract in Wistar rats. Elevated ALT and leukocytes at high doses indicate dose‐dependent systemic responses. The results showed Elevated ALT and leukocytes at high doses with sporadic mortality in 2000 and 10000 mg/Kg doses only indicate dose‐dependent systemic responses. The absence of mortality does not necessarily mean the absence of biological effects. Although the extract exhibited a favorable safety profile under the experimental conditions employed, the present findings should be interpreted within the context of the tested species and administration routes. Previous studies have demonstrated that toxicological outcomes may vary substantially between oral and intraperitoneal administration due to differences in absorption, bioavailability, and first‐pass metabolism [71, 72]. Furthermore, species‐specific physiological differences may influence toxicological responses. Therefore, the current biosafety conclusions are limited to the models and exposure routes investigated, and additional studies employing alternative administration routes and long‐term toxicity assessments are warranted.

## 5 Conclusion

Collectively, these findings demonstrate that the *S. gregaria* methanolic extract possesses notable in vitro antioxidant and cytotoxic properties, coupled with a favorable safety profile in the tested organisms. These results suggest promising therapeutic potential for the management of oxidative stress‐related degenerative diseases. Nevertheless, further investigations involving different organisms, chronic toxicity studies, and alternative routes of administration are warranted to fully elucidate its therapeutic applicability and safety margin.

## Author Contributions

M.M.A., I.M.E.‐G., and S.H.M., designed the project. R.M.K., and H.M.R.H. performed the experiments. S.H.M., S.M.S., R.M.K., and H.M.R.H. analyzed the data and interpreted the data. S.H.M., S.M.S., R.M.K., and H.M.R.H. edited and reviewed the manuscript. All authors have read and agreed to the published version of the manuscript.

## Funding

The authors have nothing to report.

## Conflicts of Interest

The authors declare no conflicts of interest.

## Ethical Approval

The Menoufia University Authority for Animal Use in Research gave its approval for this work (MUFS/F/GE/825).

## Declaration of Generative AI and AI‐Assisted Technologies in the Writing Process

During the preparation of this work the authors used ChatGpt in order to improve language and readability of the manuscript. After using this tool/service, the authors reviewed and edited the content as needed and take full responsibility for the content of the publication.

## Supporting information




**Supporting File 1**: cbdv71685‐sup‐0001‐tableS1.docx

## Data Availability

Data are available upon request from the corresponding author.
