# Supplementary material for: Antioxidant Potential of Schistocerca gregaria (Orthoptera: Acrididae) Extract: HPLC Analysis, Molecular Docking, and Bioassays
Source: Chem Biodivers. 2026 Sep 23;23(9):e71685. doi: 10.1002/cbdv.71685 (PMC13599466; doi:10.1002/cbdv.71685)
Supplement: Supplementary file 1 [file CBDV-23-e71685-s001.docx]

Table (S1): Extra Sum -of- squares-F-test comparisons of IC_50_/EC_50_ values for DPPH, ABTS, and FRAP assays among nymphal instars and ascorbic acid.

| Assay | Comparison | IC_50/_ EC_50_ | F | DFn | DFd | p-value |
| --- | --- | --- | --- | --- | --- | --- |
| DPPH | 2^nd^ stage vs 5^th^ stage | 191.5 vs. 27.73 | 1746 | 1 | 62 | <0.0001 |
| DPPH | 3^rd^ stage vs 5^th^ stage | 76.57 vs. 27.73 | 246.5 | 1 | 62 | <0.0001 |
| DPPH | 4^th^ stage vs 5^th^ stage | 94.66 vs. 27.73 | 481.5 | 1 | 62 | <0.0001 |
| DPPH | 2^nd^ stage vs ascorbic acid | 191.5 vs.7.415 | 3656 | 1 | 62 | <0.0001 |
| DPPH | 3rd stage vs ascorbic acid | 76.57 vs. 7.415 | 1079 | 1 | 62 | <0.0001 |
| DPPH | 4^th^ stage vs ascorbic acid | 94.66 vs. 7.415 | 1636 | 1 | 62 | <0.0001 |
| DPPH | 5^th^ stage vs ascorbic acid | 27.73 vs. 7.415 | 258.7 | 1 | 62 | >0.0001 |
| ABTS | 2^nd^ stage vs 5^th^ stage | 254 vs. 34.32 | 2407 | 1 | 62 | <0.0001 |
| ABTS | 3^rd^ stage vs 5^th^ stage | 127.8 vs. 34.32 | 656.6 | 1 | 62 | <0.0001 |
| ABTS | 4^th^ stage vs 5^th^ stage | 187.3 vs. 34.32 | 1551 | 1 | 62 | <0.0001 |
| ABTS | 2^nd^ stage vs ascorbic acid | 254 vs. 13.95 | 3907 | 1 | 62 | <0.0001 |
| ABTS | 3^rd^ stage vs ascorbic acid | 127.8 vs. 13.95 | 1547 | 1 | 62 | <0.0001 |
| ABTS | 4^th^ stage vs ascorbic acid | 187.3 vs. 13.95 | 2860 | 1 | 62 | <0.0001 |
| ABTS | 5^th^ stage vs ascorbic acid | 34.32 vs.13.95 | 258.7 | 1 | 62 | <0.0001 |
| FRAP | 2^nd^ stage vs 5^th^ stage | 611.3 vs. 74.71 | 2056 | 1 | 62 | <0.0001 |
| FRAP | 3^rd^ stage vs 5^th^ stage | 201.8 vs. 74.41 | 324.9 | 1 | 62 | <0.0001 |
| FRAP | 4^th^ stage vs 5^th^ stage | 273.1 vs. 74.41 | 821.1 | 1 | 62 | <0.0001 |
| FRAP | 2^nd^ stage vs ascorbic acid | 611.3 vs. 19.92 | 4967 | 1 | 62 | <0.0001 |
| FRAP | 3^rd^ stage vs ascorbic acid | 201.8 vs. 19.92 | 1788 | 1 | 62 | <0.0001 |
| FRAP | 4^th^ stage vs ascorbic acid | 273.1 vs. 19.92 | 3286 | 1 | 62 | <0.0001 |
| FRAP | 5^th^ stage vs ascorbic acid | 74.41 vs. 19.92 | 615.4 | 1 | 62 | <0.0001 |
